# Oat Protein Concentrates with Improved Solubility Produced by an Enzyme-Aided Ultrafiltration Extraction Method

**DOI:** 10.3390/foods10123050

**Published:** 2021-12-08

**Authors:** Mika Immonen, Julia Myllyviita, Tuula Sontag-Strohm, Päivi Myllärinen

**Affiliations:** 1 Department of Food and Nutrition, Faculty of Agriculture and Forestry, University of Helsinki, P.O. Box 27, FI-00014 Helsinki, Finland; Tuula.sontag-strohm@helsinki.fi; 2 Valio Ltd., P.O. Box 10, FI-00039 Helsinki, Finland; Paivi.Myllarinen@valio.fi; 3 Department of Industrial Energy Processes and Sustainability, Faculty of Advanced Energy Solutions, Aalto University, P.O. Box 16100, FI-00076 Aalto, Finland; myllyviitajulia@gmail.com

**Keywords:** oat protein concentrate, deamidation, heat-induced gelation, protein-glutaminase, ultrafiltration, diafiltration

## Abstract

The aim of this study was to develop an extraction method to produce highly functional oat protein concentrates. We investigated the possibility of combining enzyme-aided slightly alkaline (pH 8.0) extraction with ultrafiltration and subsequent diafiltration for concentration of the extracted oat proteins. A further aim was to study how the deamidation of oat proteins with protein-glutaminase (PG) improves the solubility of proteins as a function of the following parameters: pH (6.0–9.0), enzyme dosage (4–20 U/g protein), and incubation time (1–4 h) with response surface methodology (RSM). Furthermore, we investigated selected functional properties, such as heat-induced gelation and solubility, of the oat protein concentrates. The chosen parameters for the enzymatic deamidation pre-treatment process by PG were as follows: pH 8.0, dosage 11.0 U/g protein, and an incubation time of 4 h (1 h at native pH and 3 h at pH 8.0). Two oat protein concentrates were produced, non-deamidated and ultrafiltered, and deamidated and ultrafiltered, with protein concentrations of 45.0 and 52.4%, respectively. The solubility of both oat protein concentrates was significantly improved at neutral and slightly alkaline pH compared to the solubility of proteins extracted from the starting material. Additionally, both oat protein concentrates produced equally strong heat-induced gel-like structures at a protein concentration of 10%.

## 1. Introduction

Oat (*Avena sativa* L.) is an interesting cereal due to its higher protein content compared to the other cereals such as wheat, barley, and rye [1]. The distribution of proteins in the layers of oat groats is as follows: 12% protein in the starchy endosperm, 18–30% in the bran, and 29–38% in the germ [2,3]. The majority (70–80%) of oat proteins are globulins. Oat globulin proteins are primarily situated in the starchy endosperm in the protein bodies and in the aleurone layer. The second most abundant protein group is the prolamins (avenins), which account for 4–15% of the total protein [4,5]. Another minor protein group in oats is the water-soluble albumins. Their proportion of the total protein varies depending on the oat source (cultivars), growth conditions, and extraction method.

Oat protein concentrates and isolates are commonly produced using alkaline extraction or dry fractionation [6,7]. The most effective procedure to extract proteins from grain material, such as oat bran, is the former [8]. The downsides of extraction in highly alkaline pH (pH above 9.0) are changes in the protein structure, such as hydrolysis of native proteins, crosslinking, and racemization of amino acids, as well as a decrease in the techno-functional properties of proteins [9,10]. Consequently, to extract proteins in their native state, a milder alkaline pH (pH 8.0) has been shown to be the more suitable approach [11]. One of the upsides of alkaline extraction is that it helps to solubilize the proteins behind the mechanical barriers created by fibers, such as β-glucans, arabinoxylans, and cellulose. However, β-glucan and arabinoxylans increase viscosity, which leads to challenges during the alkaline extraction process, subsequently decreasing the yield of extractable protein [8,12]. In addition, starch pasting increases viscosity, if heat is required during the extraction process. These barrier components can be hydrolyzed enzymatically using carbohydrate degrading enzymes; thus, enhancing the extractability of proteins [13,14]. Therefore, enzymatically assisted extraction is an effective way to increase the protein extractability from grain materials.

It is known that oat proteins have limited solubility under neutral or slightly acidic pH, which are the most common pH conditions in food systems [15]. The functionality of oat proteins has been improved using enzymatic and chemical modifications, such as succinylation and acetylation [16], deamidation with enzymes or with acids [17,18], and hydrolysis with proteolytic enzymes [19]. Enzymatic deamidation of oat proteins with protein-glutaminase (PG, purified from *Chryseobacterium proteolyticum*, EC.3.5.1.44) as a pre-treatment could be a potential approach to increase the extraction yield when extracting proteins with alkaline extraction (pH > 9.0). PG hydrolyzes the side chain amino groups of specific protein-bound amino acid residues, to release ammonia [20]. The reaction occurs on the glutamine and asparagine residues of proteins, to make glutamic and aspartic acid, respectively. For example, Jiang et al. [17] showed that deamidation with PG did not significantly alter the structure of oat proteins (i.e., it did not cause proteolysis). To the best of our knowledge, PG has not been utilized as a pre-treatment for extracting proteins from oats. Furthermore, the combination of enzymes, β-glucanase, α-amylase, and amyloglucosidase has not been utilized to enhance the extractability of oat proteins in combination with slight alkaline extraction (pH 8.0). Moreover, only two studies have investigated the application of ultrafiltration to concentrated oat proteins and used highly alkaline pH (pH > 9.0) in the protein extraction process [21,22].

This study aimed to develop an extraction method for producing oat protein concentrates with improved techno-functional properties; as well as the possibility of combining enzyme-aided slightly alkaline (pH 8.0) extraction with ultrafiltration and subsequent diafiltration for the concentration of the extracted oat proteins. Additionally, a further aim was to investigate deamidation with PG and how this pre-treatment improves the solubility of oat proteins, as a function of the following parameters: pH (6.0–9.0), enzyme dosage (4–20 U/g protein), and incubation time (1–4 h). To reduce the number of experiments, a response surface methodology (RSM) was utilized in this study. Furthermore, we investigated selected techno-functional properties, such as heat-induced gelation and solubility, of the oat protein concentrates.

## 2. Materials and Methods

### 2.1. Materials

Commercial organic oat flour was obtained from Fazer Mills (Lahti, Finland). Alpha-amylase BAN480L (activity 480 KNU-B/g) was obtained from Novozymes (Rotterdam, The Netherlands). Amyloglucosidase Amigase Mega L (activity 36,000 AGI/g) andβ-glucanase Filtrase NL Fast (activity 40,000 BFG/g) were obtained from DSM (Delft, The Netherlands). Protein-glutaminase PG500 (activity 500 U/g) was obtained from Ajinomoto (Tokyo, Japan).

### 2.2. Optimization of Protein-Glutaminase Deamidation and Protein Solubilization

The sample composition for the optimization trials was as follows: the oat flour content of the suspension was 20% of total mass, and the content of the enzymes α-amylase, β-glucanase, and amyloglucosidase was 0.1% of the total mass. The amount of oat flour and enzymes was based on earlier experiments and kept fixed in all tests. The oat slurry samples (with a consistent total mass of 200 g) were prepared by first adding the enzymes to water and mixed using a magnetic stirrer, followed by the addition of the oat flour. The samples were vigorously mixed by hand to create a homogenous mixture, and then the samples were heated to 60 °C in a water bath and kept for 1 h under constant mixing with a magnetic stirrer. The pH of the samples was then adjusted to the target pH value (Table 1) with 1 M HCl or 0.1 M NaOH. After this, the target amount of PG was added, and the water amount was adjusted to a total mass of 200 g. The samples were stirred at 60 °C for a target length of time (1–4 h). After incubation, the samples were centrifuged (20 °C, 4000× *g*) (Beckman Model J-6M Induction Drive Centrifuge, Beckman Instruments Inc., London, UK) for 10 min, and the samples were cooled down to approximately room temperature by the temperature-controlled centrifuge during centrifugation. The supernatants containing the soluble protein were collected and stored in the refrigerator for further analysis. A control sample was prepared correspondingly, incubated for one hour, and thereafter centrifuged, lacking the pH adjustment and protease incubation time. The protein–glutaminase (PG) dosages were calculated according to Equation (1), using the estimated protein content of oat–water suspension, PG activity, and target PG concentration as variables.

(1) $$\mathrm{Dosage}\;\left[g\right]=\frac{\mathrm{PG}\;\mathrm{target}\;\mathrm{concentration}\;\left[\frac{U}{g}\right]}{\mathrm{PG}\;\mathrm{activity}\;\left[\frac{U}{g}\right]}\times \mathrm{protein}\;\mathrm{in}\;\mathrm{sample}\;\left[g\right]$$

A face-centered composite design was used to determine the optimum conditions for the deamidation of oat protein by PG treatment. This was evaluated using a response surface method (RSM) with three factors (independent variables), including PG incubation time (1–4 h) (*x*_1_), pH (6.0–9.0) (*x*_2_), and PG dosage (4–20 U/g protein) (*x*_3_) as variables. A model was designed to simulate PG pre-treatment subsequent to a 1-h enzyme-assisted pre-treatment, hence the first enzymatic treatment was taken into account in the responses. The experiment was designed in triplicate, with 14 runs and 3 center points resulting in 17 design points (Table 1). The average values of the triplicates were used to generate an RSM model of 17 design points. The deamidation degree (DD) (*Y*_1_) and the protein solubility (PS) (*Y*_2_) were used as dependent variables (response variables). The response variables were fit to a second-order polynomial equation for each response, to obtain the coefficients (Equation (2)):

(2) $$Y=\beta_{0}+\overset{3}{\underset{i=1}{\sum }}\beta_{i}x_{i}+\overset{3}{\underset{i=1}{\sum }}\beta_{ii}x_{i}{}^{2}+\overset{2}{\underset{i=1}{\sum }}\overset{3}{\underset{j=i+1}{\sum }}\beta_{ij}x_{i}x_{j}$$

where *Y* is the response variable, *x_i_* and *x_j_* are the scaled independent variables, and *β_0_*, *β_i_, β_ii_*, and *β_ij_* are the regression parameters of variables for intercept, linear, quadratic, and interaction regression terms, respectively.

### 2.3. Deamidation Degree and Protein Solubility

The deamidation degree (DD) was calculated according to the method of Yong et al. [23]. DD was expressed as the ratio of ammonia produced by the deamidation by PG treatment to the ammonia produced in a total deamidation reaction (Equation (3)), due to the stoichiometric relation between the amount of reacted amino acid residues and the ammonia produced.

(3) $$\mathrm{DD}\;\;\left[\%\right]=\frac{\mathrm{Ammonia}\;\mathrm{after}\;\mathrm{protease}\;\mathrm{treatment}\;\left[\frac{g}{L}\right]}{\mathrm{Ammonia}\;\mathrm{after}\;\mathrm{total}\;\mathrm{deamidation}\;\left[\frac{g}{L}\right]}\times 100\;$$

The centrifuged supernatant was treated with the protein precipitating agent, trichloroacetic acid (TCA), to stop the enzymatic reaction. The sample (250 µL) was pipetted into Eppendorf tubes, together with 1 mL chilled (approximately 5 °C) 0.3 M TCA. The supernatant from the centrifuged oat–water suspension (without the PG treatment) was used as a control sample.

The ammonia released during total deamidation was evaluated by HCl treatment, using the method presented by Jiang et al. [17], with minor changes. Oat flour (400 mg) was suspended in 1.6 mL of 10% HCl and heated at 100 °C for 3 h, with mixing by hand every 20 min. The hydrolyzed oat flour samples were precipitated with TCA, equivalently to the other samples. The samples were then centrifuged (Centrifuge 5424, Eppendorf, Germany) at room temperature at 12,700× *g* for 5 min. The concentration of ammonia in the collected supernatants was measured using a commercial ammonia assay kit (Thermo Scientific, Vantaa, Finland).

The protein solubility of the supernatants (solubilized oat proteins from the oat flour suspensions) was determined by measuring the amount of nitrogen in the centrifuged supernatant. Nitrogen content was determined by the Kjeldahl method, according to the method ISO 8968-1:2014. The protein content was calculated with a conversion factor, F, of 6.25. The protein solubility (%) was calculated using Equation (4). Each experiment was replicated three times. For the verification of this model an additional experiment was performed using the following parameters: pH 8.0, incubation time of 3 h, and PG dosage of 12 U/g protein. The extracted protein and deamidation degree were determined in duplicate (in total of four samples for PS and eight samples for DD). The verification experiment values were then compared to the predicted protein solubility (%) and deamidation degree (%) values, to determine the validity of the model.

(4) $$\mathrm{Protein}\;\mathrm{solubility}\;\left[\%\right]=\frac{\mathrm{Nitrogen}\;\mathrm{in}\;\mathrm{the}\;\mathrm{supernatant}\times F}{\mathrm{Nitrogen}\;\mathrm{in}\;\mathrm{the}\;40\;g\;\mathrm{flour}\;\mathrm{sample}\;\times \;F\;}\times 100\;$$

### 2.4. The Batchwise Enzyme-Aided Extraction of Oat Proteins with Optimized Deamidation Parameters in a Pilot-Scale

The oat protein extraction was carried out at a pilot-scale (total feed of 40 kg) with the optimized parameters. Two 20-kg oat–water suspension batches were prepared, similarly to in Section 2.2, apart from that PG was added together with other enzymes at the start. The first incubation was performed at pH 6.5 at 60 °C for 1 h in a water bath, and a rotor mixer with an attached mixing blade was used to keep the oat–water suspension under constant mixing. After this, the pH was adjusted to pH 8.0 with 10% NaOH, and the suspensions were further incubated for 3 h at 60 °C. The suspensions were centrifuged at 4000× *g* (Beckman Model J-6M Induction Drive Centrifuge, Beckman Instruments Inc., London, UK) for 10 min at 25 °C. The supernatants were collected and heat-treated (75 °C; 5 min) in a water bath under constant mixing to inactivate the enzymes (protein-glutaminase, β-glucanase, and amyloglucosidase). The α-amylase remained active, due to it being heat-stable at 75 °C. After the heat treatment, the supernatants were placed overnight in a refrigerator to cool down.

The supernatants, which contained the soluble protein and other soluble components, were concentrated batch-wise using a pilot-scale ultrafiltration plant with a 50-L feed vessel that was equipped with 2.5″ polymeric polyethersulfone spiral wound membrane element with a 10 kDa molecular weight cut-off and 2044 m^2^ surface area (Synder Filtration model ST3B, Vacaville, CA, USA). Throughout the filtration process the conditions were kept constant; the temperature at 50 °C, transmembrane pressure at 1.6 bar, and the pressure difference across the element at 0.8 bar. The concentration was continued until a volumetric concentration of 2.7 was reached, measured by constantly weighing the accumulated permeate.

In order to further reduce the content of sugars and other small-weight components in the concentrate (retentate), the concentrate was rinsed with water (i.e., diafiltered). The diafiltration was performed by adjusting the feed volume of suspension to its initial volume with >40 °C tap water and re-concentrating the diluted feed until an equal amount of permeate was collected. The amount of diawater to the initial feed was in a ratio of 3.2:1.

The concentrated oat protein retentate was spray-dried (Buchi, mini spray dryer B-290, Flawil, Switzerland) at an inlet temperature of 165 °C and outlet temperature of 100 °C. The dried oat protein concentrates (DE-UF-OPC and UF-OPC) were stored in a double sealed plastic bag until use. Additionally, the extraction process was carried out to produce non-deamidated oat protein concentrate (UF-OPC). Furthermore, during the incubation phase of the pilot-scale process, samples were collected to determine the deamidation degree and protein solubility, as a function of incubation time (1–4 h), with methods presented in Section 2.3.

### 2.5. Characteristics and Functionality of Oat Protein Concentrates

#### 2.5.1. Proximate Composition of the Raw Material and Oat Protein Concentrations

The protein content (total nitrogen × 6.25) was determined using the Kjeldahl method, according to the method ISO 8968-1:2014. Ash, lipids, and moisture content were determined according to the methods ISO 8070:2007, ISO 1735:2004, and IDF26A:1993, respectively. The starch and glucose content of oat flour, DE-UF-OPC, and UF-OPC were analyzed with a commercial total starch assay kit (Amyloglucosidase/α-amylase method) from Megazyme (Bray, Ireland) and glucose assay kit (D-glucose method) from Thermo Fisher Scientific (Vantaa, Finland), respectively. The starch and glucose contents were analyzed according to the manufacturer’s instructions. The glucose content was analyzed as a duplicate measurement, in total of four samples.

#### 2.5.2. Characterization of Proteins

The oat protein DE-UF-OPC, UF-OPC, and oat flour samples were characterized with sodium dodecyl sulfate polyacrylamide gel electrophoresis (SDS-PAGE) fractionation, using 10% Bis-Tris gel Criterion™ XT, BIO-RAD, Hercules, CA, USA, with non-reduced and reduced samples. The used running buffer in the SDS-PAGE analysis was 10% 3-(N-Morpholino) propane sulfonic acid (MOPS), and the reducing agent was mercaptoethanol. SeeBlue Plus 2 Pre-stained protein standard (Thermo Fischer Scientific, Invitrogen, Carlsbad, CA, USA) was used as a molecular weight marker, and protein bands were stained with Coomassie brilliant blue.

#### 2.5.3. Protein Solubility

The solubility of protein in DE-UF-OPC, UF-OPC, and oat flour (the starting raw material), as a function of pH (pH 2.0–10.0), was determined with a Bio-Rad DC protein assay (Bio-Rad, Hercules, CA, USA). The preparation of samples was done according to the method by Rosa-Sibakov et al. [24], with slight modifications. The samples were suspended (8% *w*/*w*) in deionized water at room temperature and mixed for 1 h with a magnetic stirrer. After this, the samples were divided into smaller portions, and the pH was adjusted between pH 2.0–10.0 in 1.0 increments using 1 M HCl or 0.1 M NaOH. After pH adjustment, the samples were stirred for another hour. Finally, samples were centrifuged at 4000× *g* for 15 min at 25 °C. After centrifugation, the samples were diluted to fit the linear interval of the bovine serum albumin protein standard curve (0.2–1.5 mg/mL protein), and the Bio-Rad DC protein assay was performed on the diluted samples, according to the manufacturer’s instructions. The samples were analyzed at 750 nm with a UV-Vis spectrophotometer (UV–1700 PharmaSpec, Shimadzu, Kyoto, Japan). Protein solubility was expressed as a percentage of the total amount of protein in the 8% *w*/*w* suspension. Each experiment was performed in duplicate.

#### 2.5.4. Preparation of Heat-Induced Gels

The gels were prepared by dispersing DE-UF-OPC and UF-OPC powders in deionized water and stirred for 60 min under constant mixing with a magnetic stirrer at room temperature. The protein content of each suspension was set to 10% *w*/*w*. The pH of the DE-UF-OPC suspension was 6.5, and the pH of the UF-OPC suspension was adjusted from pH 7.0 to 6.5 using 2% HCl. The suspension was placed in a cylindrical stainless-steel vessel (5.0 cm in height and 3.0 cm in width), which was coated with food-grade silicone oil. Nitrogen gas was used to remove air from the OPC suspensions before the vessel was sealed airtight. The vessel was immersed in an oil bath (MGW Lauda C6 CS, Baden-Württemberg, Germany) at 120 °C for 60 min. After the heat treatment, the vessel was placed in an ice bath for 5 min, before being stored overnight at 6 °C.

#### 2.5.5. Rheological Measurements

To determine the strength of the heat-induced gels the gel containing vessels were taken out from the cold storage 1 h before the measurement. The OPC gels (4 cm in height) were gently removed from the vessels and cut to slices 2 mm in height, using a cheese wire cutter. The gel slices were measured by amplitude sweep using a Anton Paar Physica MCR301 rheometer (Anton Paar GmbH, Graz, Austria). The measurement was performed using a 25-mm plate and plate geometries with a 2 mm gap. The De-UF-OPC and UF-OPC gels were measured using strain values from 0.01 to 100% and an angular frequency (*ω*) of 10 rad/s. The reported result was taken from a strain value of 0.1%. Each measurement was replicated three times and contained in itself a triplicate measurement, resulting in a total of nine measurements per sample.

### 2.6. Statistical Analysis

All results were expressed as an average of at least triplicate measurement, with error values according to the mean standard deviation, if not otherwise mentioned. MODDE^®^ Pro software version 12.0 (MKS Instrument AK, Andover, MA, USA) was used to generate the prediction models and calculate regression coefficients of the models and create the surface and contour plots. One-way ANOVA was used for statistical analysis of the gel strength of oat proteins, and this was followed by Tukey’s honestly significant difference (HSD) with *p* < 0.05. Statistical analysis was performed using Minitab Statistical Software v. 20.1.1 (Minitab, Inc., State College, PA, USA).

## 3. Results and Discussion

### 3.1. Optimization of PG Deamidation and Protein Solubility of Oat Protein

The RSM method data was obtained from 17 design points, with three independent variables (time, pH, and PG dosage) and the corresponding responses, DD and PS (Table 1). Second-degree polynomial regression models were used to express the DD (*Y*_1_) and PS (*Y*_2_) as a function of the independent variables, according to Equations (5) and (6), respectively. Response *Y*_2_ was transformed to logarithmic.

(5) $$Y_{1}=36.09430+4.310x_{1}-4.820x_{2}+8.950x_{3\;}-9.33019x_{2}{}^{2}-7.98019x_{3}{}^{2}\;+2.53750x_{1}x_{2}$$

(6) $$\;\;\;\;\;\;\;Y_{2}=1.84345+0.03660x_{1}+0.09009x_{2}+0.03359x_{3}-0.02462x_{1}{}^{2}\;-0.06517x_{2}{}^{2}+0.01141x_{1}x_{2}$$

The quality of fit, expressed by the multiple coefficients of determination R^2^, was 0.968 and 0.967 for responses DD and PS, respectively (Table 2). Meaning that over 96% of both responses could be explained by the fit model. Response predictability, Q^2^, was 0.904 and 0.913 for the regression models. The *p*-values of all the linear terms were significant (*p* < 0.05). For both DD and PS linear terms were significant (*p* < 0.05). In the case of DD quadratic terms, *x*_3_ × *x*_3_, *x*_2_ × *x*_2_, and *x*_1_ × *x*_3_ were significant (*p* < 0.05), and for PS, the quadratic term *x*_2_ × *x*_2_ was significant (*p* < 0.05).

**Figure 1:**
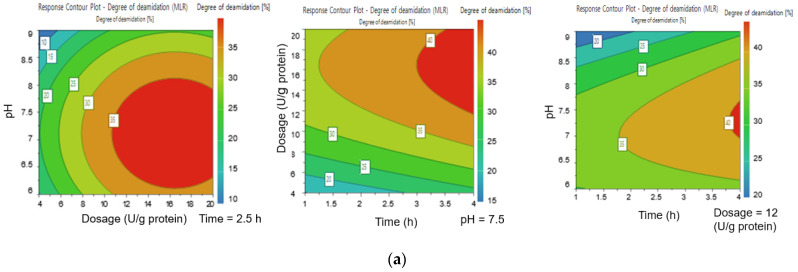

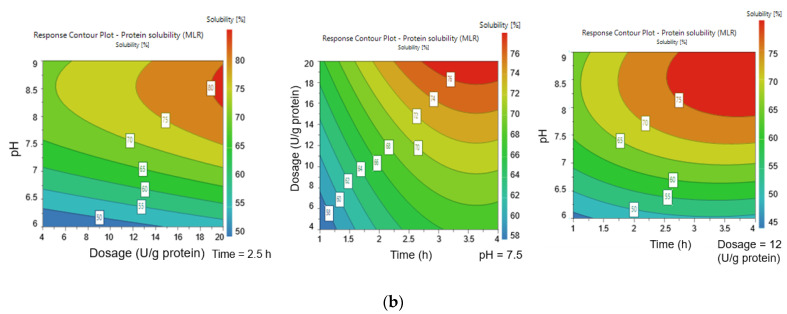
(**a**) Contour plots for deamidation degree (DD): on the left side is the pH as a function of dosage (U/g protein) with incubation time as a constant (2.5 h), in the middle is the dosage (U/g protein) as a function of time with pH as a constant (pH 7.5), and on the right side is the pH as a function of time (h), with dosage being constant (12 U/g protein). (**b**) Contour plots for protein solubility (PS): on the left side is the pH as a function of dosage (U/g protein) with incubation time as a constant (2.5 h), in the middle is the dosage (U/g protein) as a function of time, with pH as a constant (pH 7.5), and on the right side is the pH as a function of time (h), with dosage being constant (12 U/g protein).

**Figure 2:**
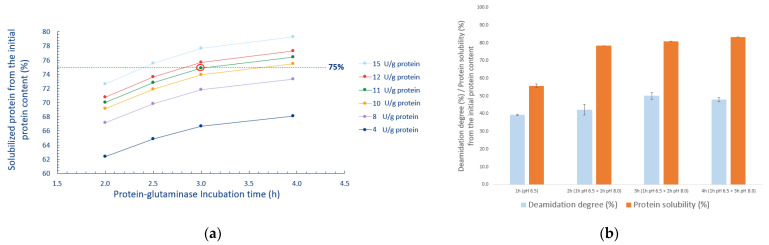
(**a**) The calculated estimations based on response surface method data of protein solubility (PS, %) as a function of incubation time, with protein-glutaminase dosages from 4 to 15 U/g protein. The dashed line (75%) is a target value, which was decided based on the data from the contour plots. (**b**) Deamidation degree (DD, %) and protein solubility (PS, %) as a function of incubation time in the pilot scale.

According to the contour plots the highest DD (40%) was reached when the PG dosage was between 16–20 U/g protein, the incubation time was 3.5 h, and pH was 7.0–8.0 (Figure 1a). The DD started to decrease when the pH increased above pH 8.0. Suppavorasatit et al. [25] reported that the highest DD of soy proteins was achieved at similar pH values, but the used PG dosage was double that used in this study. Additionally, they reported that the DD declined with pH values higher than 7.5 and lost all activity after pH 8.5. Based on our results, around 10 to 20% DD was achieved even with a high pH value of 9.0. Similar DD values were reported for coconut protein by Kunarayakul et al. [26], but only when the incubation temperature was increased above 46 °C. This indicates that PG deamidation is affected by factors such as protein type, processing conditions, and protein structure [27,28]. The highest PS (83.0%) was reached at pH 9, a PG dosage of 20 U/g protein, and incubation time of 4 h (Figure 1b), these were the optimum extraction parameters according to the RSM data. The suitability of this model was tested by an additional independent experiment using the following parameters: pH 8.0, incubation time of 3 h, and PG dosage of 12 U/g protein. The results indicated that the experimental PS (75.8 ± 0.4%) and DD (35.0 ± 1.7%) were not significantly different from the predicted PS value of 75.3% and DD value of 35.2%. The lowest PS (40.8%) was at pH 6, a PG dosage of 4 U/g protein, and incubation time of 1 h. The higher PS at alkaline pH (pH > 8.0) was impacted more by the quadratic effect of pH than deamidation with PG. It was noticed that the PS increased as a function of pH, even though the DD decreased (Supplementary Figure S1). This increase in PS as a function of pH is in accordance with other studies, where increasing the extraction pH from 6.0 to 9.0 significantly improved the solubility of oat proteins [29,30]. This can be explained by an increased net charge of proteins, away from the isoelectric point (IEP) [31]. However, it has been reported that using high alkaline pH (above pH 9.5) in the extraction process can lead to the partial denaturation of proteins and alteration of amino acids, leading to the formation of compounds such as lysinoalanine [32,33]. On the other hand, slightly alkaline pH (pH 8.0) has been shown to stabilize oat lipids from oxidation [34]. Additionally, the RSM method data was used to simulate how PG dosages from 4 to 15 U/protein increase the solubility of oat proteins, as a function of incubation time at pH 8.0 (Figure 2a). Based on this, it was concluded that 75% PS would be achieved with a dosage of 11 U/g protein, which corresponded to the highest PS in two of the contour plots in Figure 1b. Moreover, considering the economic feasibility of this extraction process, it would be advisable to utilize a moderate amount of enzyme preparation. Taking results from the optimization trials into consideration, the parameters were chosen for the pilot scale extraction process were as follows: incubation time of 1 + 3 h, pH 8.0, and PG dosage of 11 U/g protein. Furthermore, due to the slight decrease in the PG enzyme activity at pH 8.0, the PG enzyme was added in the start of the extraction process.

### 3.2. The Batchwise Enzyme-Aided Extraction of Oat Proteins at Pilot-Scale

The DD and PS were determined as a function of incubation time (Figure 2b). Higher PS (around 80%) and DD (above 45%) were achieved due to the addition of PG enzyme at the start of the process. Therefore, the values of PS and DD were higher than the RSM method model predicted. Jiang et al. [17] reported a DD of 59% when using higher PG dosages (26 U/g) and longer incubation times (12 h) at pH 7.0. Additionally, they studied DD% as a function of incubation time, with a PG dosage of 13 U/g, and reported a similar DD of more than 40% when the incubation time was less than 5 h. For the case of soy proteins, Suppavorasatit et al. [25] reported a DD of about 40% and PS of 70%, with an incubation time of about 2.0 h and PG dosage of 40 U/g protein.

The concentration of starch in the oat protein concentrates was decreased from 49.0% to approximately 2.5% by the enzymes that were utilized in the enzyme-aided extraction process (Table 3). Oat protein concentrates DE-UF-OPC and UF-OPC were successfully produced using the enzyme-aided extraction method with ultrafiltration concentration and subsequent diafiltration at pilot-scale. DE-UF-OPC (52.4%) had a significantly higher (*p* < 0.05) protein concentration than UF-OPC (45%), suggesting that deamidation improved the extractability of oat proteins. Oat protein concentrates that have been produced using higher alkaline pH (pH >9.0) typically have a protein concentration of 60 to 70% [30,35]. However, the extracted protein yields with alkaline extraction (pH >9.0) can be as low as 10 to 15% of the total protein content [36,37]. The oat protein concentrates produced in this work were extracted using lower pH values, which can decrease the protein content in the protein concentrates. The yield of proteins can be increased with the assistance of enzymes. For example, Guan et al. [37] reported a significant increase in the amount of extracted protein with an enzyme-aided extraction method (yield of 56.2%) using Viscozyme L (contains several carbohydrate hydrolyzing enzymes), a significant improvement when compared to the alkaline extraction and acid precipitation method, with a yield of 14.8% of total proteins. Furthermore, Jodayree et al. [8] used α-amylase or amyloglucosidase enzymes as a pre-treatment in combination with a highly alkaline pH and acid precipitation to extract proteins from defatted oat bran. They reported that the protein extractability was increased from 50% to 72% and 80% by using α-amylase and amyloglucosidase, respectively.

### 3.3. Characterization of Oat Proteins

**Figure 3:**
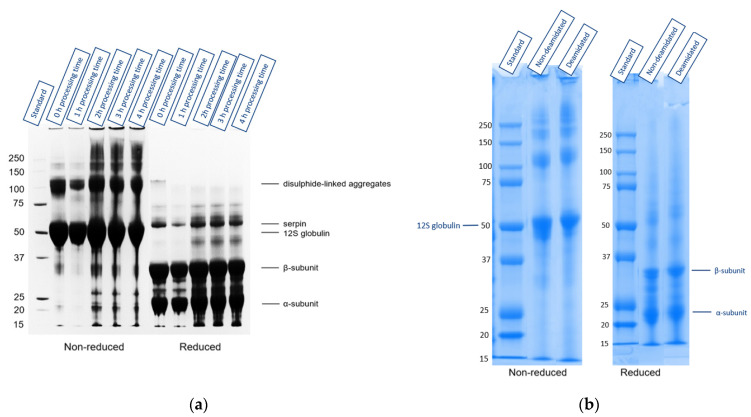
(**a**) Non-reduced and mercaptoethanol-reduced 10% of Bis-Tris gel with indications of the samples as a function of the processing time, starting from 0 to 4 h, and a molecular weight marker (standard). (**b**) Non-reduced and mercaptoethanol-reduced samples of 10% Bis-Tris gel with indications of the samples molecular weight (Standard), deamidated and ultrafiltrated oat protein concentrate (DE-UF-OPC), and ultrafiltrated oat protein concentrate (UF-OPC).

SDS-PAGE was undertaken to determine whether the structure of oat proteins was changed during the extraction process (Figure 3a). Furthermore, SDS-PAGE was performed from the DE-UF-OPC and UF-OPC, to determine if there were any noticeable differences between the oat protein concentrates (Figure 3b). There was a slight, but noticeable, shift in the DE-UF-OPC protein bands compared to the UF-OPC, which was most probably due to the deamidation of oat proteins. The main oat protein band was approximately 50 kDa in the non-reduced SDS-PAGE, in both DE-UF-OPC and UF-OPC (Figure 3b). Under reducing conditions, the 30 kDa and 20 kDa bands were the most dominant in both SDS-PAGEs. Walburg and Larkins [38] reported the 50 kDa band as a native oat globulin protein that breaks into 30 kDa and 20 kDa subunits in reducing conditions. These results were in agreement with Immonen et al. [39], who used SDS-PAGE to characterize the structure of destarched oat protein concentrate.

### 3.4. Solubility of the Oat Protein Concentrates as a Function of pH

**Figure 4:**
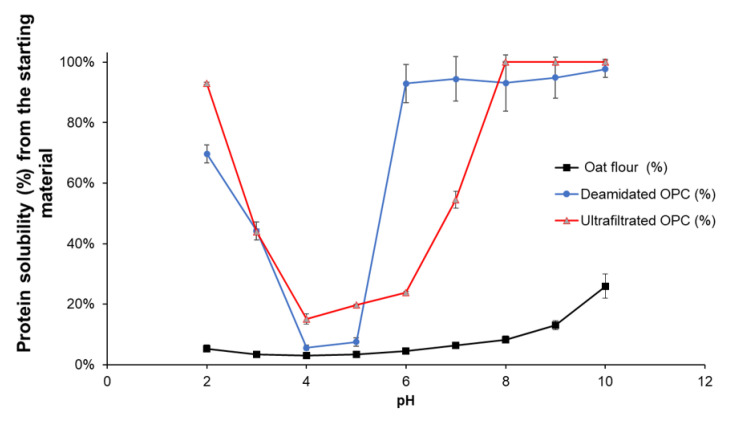
Protein solubility of oat flour (starting material), deamidated and ultrafiltrated oat protein concentrate (DE-UF-OPC), and ultrafiltrated oat protein concentrate (UF-OPC) as a function of pH (2.0–10.0).

The PS of oat flour (starting material), DE-UF-OPC, and UF-OPC as a function of pH is presented in Figure 4. The PS of DE-UF-OPC was over 90% at pH 6.0, which is a remarkable improvement in protein functionality in comparison to that of oat flour. Jiang et al. [17] reported that due to deamidation by PG, negatively charged carbonyl side-chains were formed, thus increasing the electronic repulsion forces between the oat protein particles. Additionally, they suggested that the possible mechanism for the improved water solubility of oat proteins was the consequence of a combination of the increased flexibility of the protein structure and increased net charge of the proteins. Additionally, UF-OPC had a good PS at pH 7.0, when pH was pH 8.0 and above, the solubility of the proteins increased to almost 100%. Surprisingly, UF-OPC had a higher protein solubility (above 90%) than DE-UF-OPC (about 70%) at acidic pH (pH 2.0). The PS curve of UF-OPC resembles the solubility curve of purified oat globulin from pH 2.0 to 10.0 [30].

### 3.5. Rheological Properties of Heat-Induced Oat Protein Concentrate Gels

**Figure 5:**
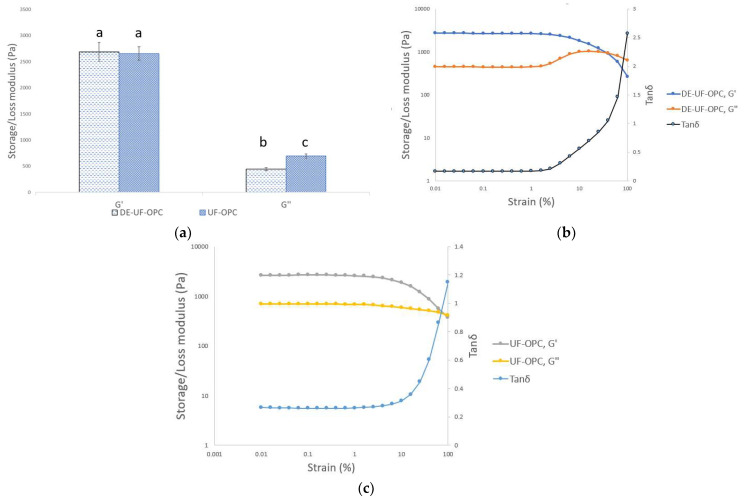
(**a**) Storage modulus (G′) and loss modulus (G″) of deamidated and ultrafiltrated oat protein concentrate (DE-UF-OPC) and ultrafiltrated oat protein concentrate (UF-OPC) at 0.1% strain. Different letters (a, b, c) indicate significant differences (*p* < 0.05) between the storage and loss modulus. (**b**) Storage modulus (G′) and loss modulus (G″) of deamidated and ultrafiltrated oat protein concentrate (DE-UF-OPC) as a function of strain (%) and right-side *y*-axis tanδ as a function of strain (%). (**c**) Storage modulus (G′) and loss modulus (G″) of ultrafiltrated oat protein concentrate (UF-OPC) as a function of strain (%) and right-side *y*-axis tanδ as a function of strain (%).

The gel strengths of DE-UF-OPC and UF-OPC were about 2700 and 2650 Pa, respectively, at a constant protein concentration of 10% and amplitude strain of 0.1% (Figure 5a). There were not significant (*p* > 0.05) differences between the storage moduli (G′) of oat protein concentrates, which indicates that the gels were equally strong at 0.1% strain. To our knowledge, no similar gel strength measurements have been published using a similar protein extraction method, but oat protein isolate manufactured by alkaline extraction and acid precipitation has been shown to produce strong heat-induced gel-like structures [40,41]. For instance, Nieto-Nieto et al. [40] studied the gelation properties of oat protein isolate using frequency sweep, which, although not directly comparable to the results presented in this work, nevertheless this gives indicative information about the gelation properties of oat proteins. However, they produced a solid oat protein heat-induced gel-like structure (pH 7.0) at 13.5% protein concentration with gel strength of 18 kPa, which was six times stronger than the DE-UF-OPC and UF-OPC gel-like structure. Additionally, Brückner-Gühmann et al. [41] produced heat-induced oat protein gels at 120 °C and pH 8.0, with a protein content of about 12.5%. They reported that the heat-induced oat protein gels with a gel strength above 20 kPa were comparable to gelatine gels (with a protein concentration of about 10%). This difference in gel strength is most likely due to the difference in the protein extraction method and purity of the studied protein. This was shown earlier by Yang et al. [42], where the selected protein extraction method had a significant impact on the gelation properties of pea protein isolates.

However, the gelation properties of DE-UF-OPC and UF-OPC gels are comparable to other protein isolates. For instance, Kornet et al. [43] studied the gelation properties of pea protein isolate (produced by alkaline extraction acid precipitation method) and concentrate, with amplitude and frequency sweep at 10% protein content. According to their amplitude sweep data the pea protein isolate formed a heat-induced gel with a gel strength below 1000 Pa. Moreover, Nivala et al. [44] studied the gelation properties of a fava bean protein isolate that was produced by alkaline extraction and acid precipitation using amplitude sweep. They reported that the gel strength (G′) of heat-induced transglutaminase treated (10 nkat/g protein) fava bean protein isolate gels at 10% protein content was below 500 Pa. When the transglutaminase dosage was increased to 100 nkat/g protein, the gel strength of the fava bean protein increased to 3500 Pa, which is slightly higher than the gel strengths of the DE-UF-OPC and the UF-OPC. Our results suggest that the oat protein concentrates that were produced with the extraction method presented in this study can produce comparable gel-like structures to the protein isolates that were produced by alkaline extraction and acid precipitation.

The loss moduli (G″) of both gels were significantly different (*p* < 0.05). The linear viscosity region (LVR) was noticeable longer in UFC-OPC, when compared to the DE-UF-OPC (Figure 5b,c). Additionally, the loss modulus (G″) of DE-UF-OPC increased at 4% strain, before crossing the storage modulus (G″). This behavior was not noticed in the UF-OPC, there was only a decrease in the loss modulus at the yield point (64% strain). Bi et al. [45] reported a similar G″ behavior for 8% soy protein isolate, when studied with strain amplitude sweep using an angular frequency from 0.1 to 3 Hz. They described this type of flow behavior as weak strain overshoot, which is the characteristic behavior for soft glassy materials, such as concentrated protein emulsions [46]. This means that when an external strain is applied, the structure resists the deformation to a certain strain, and this is the point where G″ increases [47]. Additionally, when the deformation increases over the critical strain (yield point), the structure is fractured and G″ starts to decrease.

## 4. Conclusions

In this work, two oat protein concentrates, with a protein concentration of about 40 to 50%, were successfully produced with an enzyme-aided slightly alkaline pH (pH 8.0) extraction method, with ultrafiltration and subsequent diafiltration concentration at pilot-scale. Additionally, the extractability of oat proteins was further enhanced by enzymatical deamidation with PG. The DE-UF-OPC had significantly improved solubility in water at neutral pH, where oat proteins typically remain insoluble. Surprisingly, the ultrafiltration process also improved the solubility of the oat proteins at a slightly alkaline pH. Furthermore, both UF-OPC and DE-UF-OPC produced solid heat-induced gel-like structures. The gel strength results of the oat protein concentrates were comparable to the gel strength results of heat-induced gel-like structures made from pea and fava bean protein isolate that are presented in the literature. This raises an interesting question of whether UF-OPC and DE-UF-OPC could be used as oat protein ingredients in plant-based food applications, such as in drinks and yogurt alternatives. There is a need for further studies on how these oat protein ingredients behave in food model systems other than solid food applications. Moreover, further studies are required on how this extraction method affects the structure of oat proteins.

## Figures and Tables

**Table 1:** The design points of the experimental design for protein deamidation degree (*Y*_1_, DD, %) and protein solubility (*Y*_2_, PS, %), where *x*_1_, *x*_2_, and *x*_3_ are time (h), pH, and PG dosage (U/g protein), respectively.

Design Points	Independent Variables						Dependent Variables	
NO.	*x*_1_	*x*_2_	*x*_3_	Time	pH	PG Dosage	DD	PS
1	−1	−1	−1	1	6	4	13.7	40.8
2	+1	−1	−1	4	6	4	15.1	47.4
3	−1	+1	−1	1	9	4	0.0	58.0
4	+1	+1	−1	4	9	4	9.7	71.1
5	−1	−1	+1	1	6	20	33.1	47.4
6	+1	−1	+1	4	6	20	35.2	49.8
7	−1	+1	+1	1	9	20	14.2	67.8
8	+1	+1	+1	4	9	20	28.3	83.3
9	−1	0	0	1	7.5	12	27.7	58.8
10	+1	0	0	4	7.5	12	43.5	74.3
11	0	−1	0	2.5	6	12	28.9	48.2
12	0	+1	0	2.5	9	12	25.6	75.2
13	0	0	−1	2.5	7.5	4	20.0	58.0
14	0	0	+1	2.5	7.5	20	37.2	75.2
15	0	0	0	2.5	7.5	12	37.5	73.5
16	0	0	0	2.5	7.5	12	35.5	71.1
17	0	0	0	2.5	7.5	12	35.3	71.9

**Table 2:** Regression coefficients for the surface response model of selected independent variables and statistical information of the deamidation degree (%) and protein solubility (%) of oat proteins.

Deamidation Degree	Coefficient	*p*-Value		
Constant	36.094	1.804 × 10^−11^	Q^2^ = 0.904	
*x*_1_ (Time)	4.3100	0.000496	R^2^ = 0.968	
*x*_2_ (pH)	−4.8200	0.000211	R^2^ adj. = 0.949	
*x*_3_ (Dosage)	8.9500	1.019 × 10^−6^		
*x*_2_ × *x*_2_ (pH × pH)	−9.3301	0.000128	Cond. no. = 3.978	
*x*_3_ × *x*_3_ (Dosage × Dosage)	−7.9801	0.000431	RSD = 2.697	
*x*_1_ × *x*_3_ (Time × Dosage)	2.5375	0.023848	Confidence = 0.95	
**Protein Solubility**	**Coefficient**	***p*-Value**		
Constant	1.8434	1.564 × 10^−19^	Q^2^ = 0.913	
*x*_1_ (Time)	0.0366	0.000300	R^2^ = 0.967	
*x*_2_ (pH)	0.0900	1.109 × 10^−7^	R^2^ adj. = 0.947	
*x*_3_ (Dosage)	0.0335	0.000572		
*x*_1_ × *x*_1_ (Time × Time)	−0.0246	0.073512 *	Cond. no. = 3.978	
*x*_2_ × *x*_2_ (pH × pH)	−0.0651	0.000351	RSD = 0.02143	
*x*_1_ × *x*_3_ (Time × Dosage)	0.0114	0.162965 *	Confidence = 0.95	

* Not significant (*p* > 0.05).

**Table 3:** The composition of oat flour (starting material), ultrafiltered oat protein concentrate (UF-OPC), and deamidated and ultrafiltered oat protein concentrate (DE-UF-OPC). Different letters (^a–h^) indicate the significant differences (*p* < 0.05) within the sample groups.

Sample	Protein (%)	Fat (%)	Ash (%)	Starch (%)	Glucose (%)	Moisture (%)
Oat flour	12.8 ± 0.1 ^a^	8.2 ± 0.1 ^d^	1.8 ± 0.0 ^g^	49.0 ± 1.1	-	8.1 ± 0.0
Ultrafiltered oat protein concentrate	45.0 ± 0.1 ^b^	27.6 ± 0.1 ^e^	2.9 ± 0.0 ^h^	2.5 ± 0.0	4.0 ± 0.0	1.7 ± 0.0
Deamidated and ultrafiltered oat protein concentrate	52.4 ± 0.6 ^c^	22.3 ± 0.1 ^f^	3.0 ± 0.1 ^h^	2.4 ± 0.2	6.7 ± 0.1	2.0 ± 0.3

## Supplementary Materials

The following are available online at https://www.mdpi.com/article/10.3390/foods10123050/s1, Figure S1: Surface response plots of the deamidation degree (DD) and the protein solubility (PS) as a function of time (h) and dosage (U/g protein) while pH being constant from 6.0 to 9.0.

## References

[1] Peterson D., Webster F., Wood P. (2011). Storage proteins. Oats Chemistry and Technology.

[2] Youngs V.L. (1972). Protein distribution in the oat kernel. Cereal Chem..

[3] Wu Y.V., Sexson K.R., Cavins J.F., Inglett G.E. (1972). Oats and Their Dry-Milled Fractions. Protein Isolation and Properties of Four Varieties. J. Agric. Food Chem..

[4] Chang Y.-W., Alli I., Konishi Y., Ziomek E. (2011). Characterization of Protein Fractions from Chickpea (*Cicer arietinum* L.) and Oat (*Avena sativa* L.) Seeds Using Proteomic Techniques. Food Res. Int..

[5] Mäkinen O.E., Sozer N., Ercili-Cura D., Poutanen K., Nadathur S.R., Wanasundara J.P.D., Scanlin L. (2017). Protein from oat: Structure, Processes, Functionality, and Nutriotion. Sustainable Protein Sources.

[6] Wu Y.V., Sexson K.R., Cluskey J.E., Inglett G.E. (1977). Protein Isolate from High-Protein Oats: Preparation, Composition and Properties. J. Food Sci..

[7] Sibakov J., Myllymäki O., Holopainen U., Kaukovirta-Norja A., Hietaniemi V., Pihlava J.M., Poutanen K., Lehtinen P. (2011). Lipid Removal Enhances Separation of Oat Grain Cell Wall Material from Starch and Protein. J. Cereal Sci..

[8] Jodayree S., Smith J.C., Tsopmo A. (2012). Use of Carbohydrase to Enhance Protein Extraction Efficiency and Antioxidative Properties of Oat Bran Protein Hydrolysates. Food Res. Int..

[9] Ruiz G.A., Xiao W., van Boekel M., Minor M., Stieger M. (2016). Effect of Extraction pH on Heat-Induced Aggregation, Gelation and Microstructure of Protein Isolate from Quinoa (*Chenopodium quinoa* Willd). Food Chem..

[10] Gao Z., Shen P., Lan Y., Cui L., Ohm J.-B., Chen B., Rao J. (2020). Effect of Alkaline Extraction PH on Structure Properties, Solubility, and Beany Flavor of Yellow Pea Protein Isolate. Food Res. Int..

[11] Tanger C., Engel J., Kulozik U. (2020). Influence of Extraction Conditions on the Conformational Alteration of Pea Protein Extracted from Pea Flour. Food Hydrocoll..

[12] Sibakov J., Myllymäki O., Suortti T., Kaukovirta-Norja A., Lehtinen P., Poutanen K. (2013). Comparison of Acid and Enzymatic Hydrolyses of Oat Bran β-Glucan at Low Water Content. Food Res. Int..

[13] Arte E., Katina K., Holopainen-Mantila U., Nordlund E. (2016). Effect of Hydrolyzing Enzymes on Wheat Bran Cell Wall Integrity and Protein Solubility. Cereal Chem..

[14] Prosekov A., Babich O., Kriger O., Ivanova S., Pavsky V., Sukhikh S., Yang Y., Kashirskih E. (2018). Functional Properties of the Enzyme-Modified Protein from Oat Bran. Food Biosci..

[15] Loponen J., Laine P., Sontag-Strohm T., Salovaara H. (2007). Behaviour of Oat Globulins in Lactic Acid Fermentation of Oat Bran. Eur. Food Res. Technol..

[16] Zhao C.-B., Zhang H., Xu X.-Y., Cao Y., Zheng M.-Z., Liu J.-S., Wu F. (2017). Effect of Acetylation and Succinylation on Physicochemical Properties and Structural Characteristics of Oat Protein Isolate. Process Biochem..

[17] Jiang Z., Sontag-Strohm T., Salovaara H., Sibakov J., Kanerva P., Loponen J. (2015). Oat Protein Solubility and Emulsion Properties Improved by Enzymatic Deamidation. J. Cereal Sci..

[18] Mirmoghtadaie L., Kadivar M., Shahedi M. (2009). Effects of Succinylation and Deamidation on Functional Properties of Oat Protein Isolate. Food Chem..

[19] Brückner-Gühmann M., Heiden-Hecht T., Sözer N., Drusch S. (2018). Foaming Characteristics of Oat Protein and Modification by Partial Hydrolysis. Eur. Food Res. Technol..

[20] Yamaguchi S., Jeenes D.J., Archer D.B. (2001). Protein-Glutaminase from *Chryseobacterium proteolyticum*, an Enzyme That Deamidates Glutaminyl Residues in Proteins: Purification, Characterization and Gene Cloning. Eur. J. Biochem..

[21] Xiaoping Z., Yinmao D., Yonggua L., Yan S., Qing R. (2010). Separation of oat protein hollow fiber ultrafiltration membrane and cleaning method. Trans. Chin. Soc. Agric. Eng..

[22] Каширскихшиpcких E., Kashirskich E., Бaбич O., Babich O., Kpигep O., Kriger O. (2019). Production Technology for Oat Protein with Advanced Physicochemical, Functional, and Technological Properties. Food Process. Tech. Technol..

[23] Yong Y.H., Yamaguchi S., Gu Y.S., Mori T., Matsumura Y. (2004). Effects of Enzymatic Deamidation by Protein-Glutaminase on Structure and Functional Properties of α-Zein. J. Agric. Food Chem..

[24] Rosa-Sibakov N., Re M., Karsma A., Laitila A., Nordlund E. (2018). Phytic Acid Reduction by Bioprocessing as a Tool To Improve the In Vitro Digestibility of Faba Bean Protein. J. Agric. Food Chem..

[25] Suppavorasatit I., De Mejia E.G., Cadwallader K.R. (2011). Optimization of the Enzymatic Deamidation of Soy Protein by Protein-Glutaminase and Its Effect on the Functional Properties of the Protein. J. Agric. Food Chem..

[26] Kunarayakul S., Thaiphanit S., Anprung P., Suppavorasatit I. (2018). Optimization of Coconut Protein Deamidation Using Protein-Glutaminase and Its Effect on Solubility, Emulsification, and Foaming Properties of the Proteins. Food Hydrocoll..

[27] Gu Y.S., Matsumura Y., Yamaguchi S., Mori T. (2001). Action of Protein-Glutaminase on α-Lactalbumin in the Native and Molten Globule States. J. Agric. Food Chem..

[28] Hamada J.S. (1992). Effects of Heat and Proteolysis on Deamidation of Food Proteins Using Peptidoglutaminase. J. Agric. Food Chem..

[29] Konak Ü.İ., Ercili-Cura D., Sibakov J., Sontag-Strohm T., Certel M., Loponen J. (2014). CO_2_-Defatted Oats: Solubility, Emulsification and Foaming Properties. J. Cereal Sci..

[30] Ma C.Y., Harwalkar V.R. (1984). Chemical Characterization and Functionality Assessment of Oat Protein Fractions. J. Agric. Food Chem..

[31] Deleu L.J., Lambrecht M.A., Van de Vondel J., Delcour J.A. (2019). The Impact of Alkaline Conditions on Storage Proteins of Cereals and Pseudo-Cereals. Curr. Opin. Food Sci..

[32] Jarpa-Parra M., Bamdad F., Wang Y., Tian Z., Temelli F., Han J., Chen L. (2014). Optimization of Lentil Protein Extraction and the Influence of Process PH on Protein Structure and Functionality. LWT Food Sci. Technol..

[33] Zhang Z., Wang Y., Dai C., He R., Ma H. (2018). Alkali Extraction of Rice Residue Protein Isolates: Effects of Alkali Treatment Conditions on Lysinoalanine Formation and Structural Characterization of Lysinoalanine-Containing Protein. Food Chem..

[34] Liukkonen K. (1994). Improvement of Lipid Stability in Aqueous Processing of Oats. Ph.D. Thesis.

[35] Dyshlyuk L.S., Izgaryshev A.V., Garmashov S.Y., Sukhikh A., Kashirskih E.V. (2017). Studying the Features of the Protein Extraction from Oat Grains. J. Pharm. Sci..

[36] Liu J., Guan X., Zhu D., Sun J. (2008). Optimization of the Enzymatic Pretreatment in Oat Bran Protein Extraction by Particle Swarm Optimization Algorithms for Response Surface Modeling. LWT Food Sci. Technol..

[37] Guan X., Yao H. (2008). Optimization of Viscozyme L-Assisted Extraction of Oat Bran Protein Using Response Surface Methodology. Food Chem..

[38] Walburg G., Larkins B.A. (1983). Oat Seed Globulin: Subunit Characterization and Demonstration of Its Synthesis as a Precursor. Plant Physiol..

[39] Immonen M., Chandrakusuma A., Sibakov J., Poikelispää M., Sontag-Strohm T. (2021). Texturization of a Blend of Pea and Destarched Oat Protein Using High-Moisture Extrusion. Foods.

[40] Nieto Nieto T.V., Wang Y., Ozimek L., Chen L. (2016). Improved Thermal Gelation of Oat Protein with the Formation of Controlled Phase-Separated Networks Using Dextrin and Carrageenan Polysaccharides. Food Res. Int..

[41] Brückner-Gühmann M., Kratzsch A., Sozer N., Drusch S. (2021). Oat Protein as Plant-Derived Gelling Agent: Properties and Potential of Modification. Future Foods.

[42] Yang J., Zamani S., Liang L., Chen L. (2021). Extraction Methods Significantly Impact Pea Protein Composition, Structure and Gelling Properties. Food Hydrocoll..

[43] Kornet R. (2021). Less Is More: Limited Fractionation Yields Stronger Gels for Pea Proteins. Food Hydrocoll..

[44] Nivala O., Nordlund E., Kruus K., Ercili-Cura D. (2021). The Effect of Heat and Transglutaminase Treatment on Emulsifying and Gelling Properties of Faba Bean Protein Isolate. LWT Food Sci Technol..

[45] Bi C., Wang L., Li D., Huang Z., Adhikari B., Chen X.D. (2017). Non-Linear Rheological Properties of Soy Protein Isolate Dispersions and Acid-Induced Gels. Int. J. Food Eng..

[46] Böni L., Rühs P.A., Windhab E.J., Fischer P., Kuster S. (2016). Gelation of Soy Milk with Hagfish Exudate Creates a Flocculated and Fibrous Emulsion- and Particle Gel. PLoS ONE.

[47] Hyun K., Kim S.H., Ahn K.H., Lee S.J. (2002). Large Amplitude Oscillatory Shear as a Way to Classify the Complex Fluids. J. Non-Newtonian Fluid Mech..

