# Supplementary material for: Oat Protein Concentrates with Improved Solubility Produced by an Enzyme-Aided Ultrafiltration Extraction Method
Source: Foods. 2021 Dec 8;10(12):3050. doi: 10.3390/foods10123050 (PMC8701216; doi:10.3390/foods10123050)
Supplement: Supplementary file 1 [file foods-10-03050-s001.zip › foods-1482289-supplementary.pdf]

Supplementary data

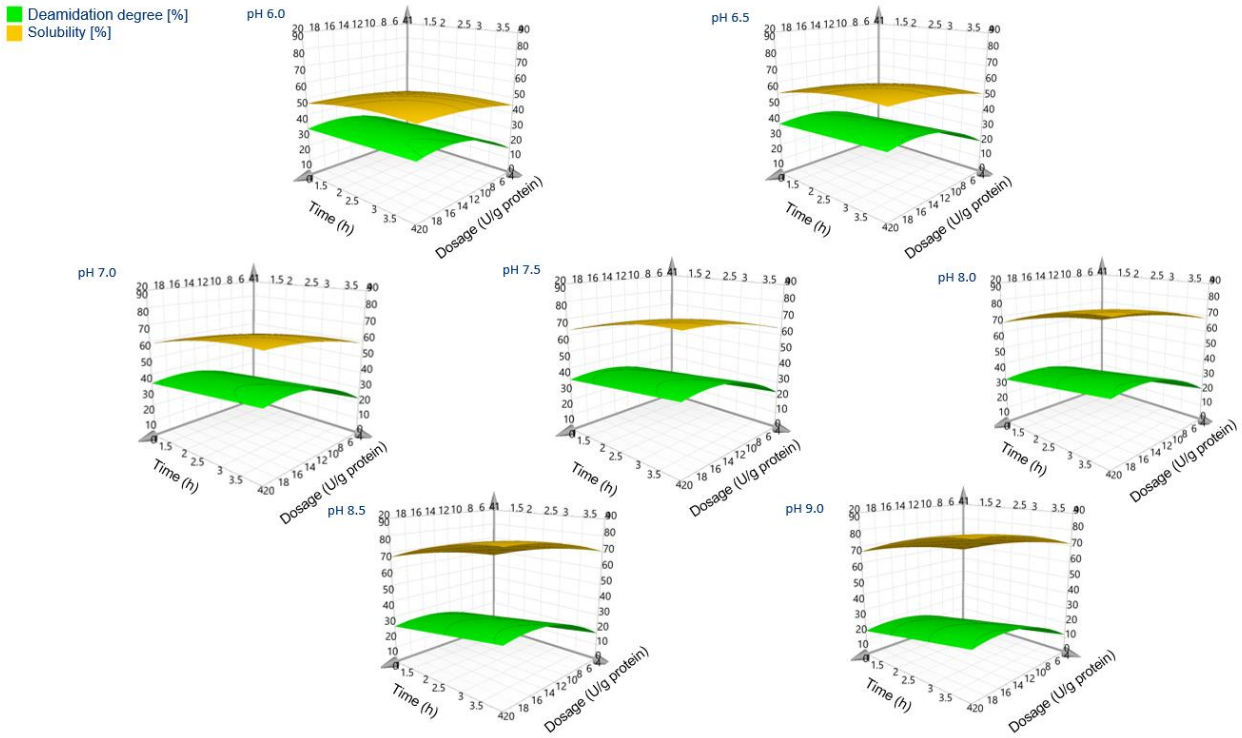

Figure S1. Surface response plots of the deamidation degree (DD) and the protein solubility (PS) as a function of time (h) and dosage (U/g protein) while pH being constant from 6.0 to 9.0.
